# Panobinostat Potentiates the Antitumor Efficacy of 5-Fluorouracil in Gastric Cancer by Suppressing Thymidylate Synthase Expression

**DOI:** 10.3390/ijms27031516

**Published:** 2026-02-03

**Authors:** Sooyeon Park, Nayeon Kim, Changwon Yang

**Affiliations:** Department of Science Education, Ewha Womans University, Seoul 03760, Republic of Korea; spark01@ewha.ac.kr (S.P.); nayeon7112@ewha.ac.kr (N.K.)

**Keywords:** gastric cancer, panobinostat, 5-fluorouracil, thymidylate synthase, histone deacetylase inhibitor

## Abstract

Resistance to 5-fluorouracil (5-FU), a cornerstone chemotherapy for gastric cancer (GC), is a major clinical obstacle, often driven by the upregulation of its target enzyme, thymidylate synthase (TS). In this study, we investigated the potential of the pan-histone deacetylase inhibitor (HDACi) panobinostat to synergize with 5-FU. In GC cell lines, panobinostat treatment alone suppressed cell viability, clonogenicity, and migration, and this was associated with the induction of G1-phase cell cycle arrest and mitochondria-mediated apoptosis. Crucially, Panobinostat acted synergistically with 5-FU, leading to enhanced cytotoxicity. Mechanistically, 5-FU treatment alone induced a compensatory upregulation of TS protein, a known resistance mechanism. Panobinostat not only suppressed basal TS expression but, more importantly, abrogated this 5-FU-induced upregulation. Furthermore, panobinostat downregulated a network of oncogenes and cell cycle regulators, including c-Myc and key cyclins. These findings indicate that panobinostat can enhance 5-FU cytotoxicity by targeting TS expression and reprogramming oncogenic transcriptional networks, supporting its potential as a complementary strategy for overcoming fluoropyrimidine resistance in GC therapy.

## 1. Introduction

Gastric cancer (GC) poses a significant global health burden, with both high incidence and mortality rates. It is the fifth-most commonly diagnosed malignancy and the fifth leading cause of cancer-related deaths worldwide [1]. While its incidence has declined, the global burden of GC is projected to increase by approximately 62% by 2040 [2], highlighting the urgent need for more effective therapeutic interventions.

Among the current treatment options, 5-fluorouracil (5-FU)-based chemotherapy remains a mainstay for patients with unresectable or recurrent GC [3]. 5-FU exerts its cytotoxic effects by inhibiting thymidylate synthase (TS) and incorporating its active toxic metabolites into RNA and DNA, disrupting nucleic acid synthesis and promoting cancer cell death [4]. However, despite its widespread use, the therapeutic efficacy of 5-FU is frequently limited by the emergence of chemoresistance [5], which remains a major clinical challenge.

The mechanisms underlying 5-FU resistance are multifaceted, involving alterations in drug metabolism, DNA repair, and cell cycle checkpoints. In particular, the overexpression of TS, encoded by the *TYMS* gene, has emerged as a key factor in acquired resistance [6,7]. TS catalyzes the de novo synthesis of thymidylate, a nucleotide essential for DNA replication. Its overexpression allows cancer cells to bypass the cytotoxic effects of 5-FU. In addition to its role as an enzyme, TS can also exhibit proto-oncogene properties, promoting tumor progression and metastasis. Clinically, elevated TS levels are associated with poor responses to 5-FU therapy and reduced overall survival in GC patients [8,9]. Furthermore, chronic 5-FU exposure may induce compensatory upregulation of TS, leading to an acquired and self-sustaining resistance phenotype [10].

Given TS’s central role in 5-FU resistance, therapeutic strategies that suppress TS expression represent a promising avenue for improving treatment efficacy [6]. Recent studies have emphasized the contribution of epigenetic regulation—particularly that of histone deacetylases (HDACs)—in regulating drug sensitivity [11,12,13]. HDACs modulate chromatin structure by removing acetyl groups from histone tails, leading to transcriptional repression of tumor suppressor genes and activation of oncogenic pathways. Importantly, HDACs have been implicated in the regulation of TS expression and 5-FU responsiveness, positioning HDAC inhibitors (HDACis) as potential therapeutic agents for overcoming chemoresistance [14].

Several HDACis, including suberoylanilide hydroxamic acid (SAHA), valproic acid, and trichostatin A, have been proven to sensitize cancer cells to 5-FU through TS suppression, cell cycle arrest, and induction of apoptosis [15,16,17]. However, most studies have focused on hematologic malignancies or other solid tumors, and their findings may not fully translate to GC, which possesses distinct epigenetic landscapes [18,19]. Among the HDACis, panobinostat—which targets class I, II, and IV HDACs—stands out as a particularly promising agent, offering broad epigenetic modulation capabilities [20]. However, its potential in GC, particularly its ability to modulate TS expression and overcome 5-FU sensitivity, remains largely unexplored [21].

In this study, we aim to investigate the therapeutic effects of panobinostat in GC cells and evaluate its potential to enhance 5-FU efficacy. We hypothesize that panobinostat sensitizes GC cells to 5-FU by downregulating TS expression and promoting apoptosis. To test this, we assessed panobinostat’s effects on cell viability, cell cycle progression, apoptosis, and mitochondrial function, both as a monotherapy and in combination with 5-FU. Furthermore, we explored the molecular mechanisms underlying TS regulation by panobinostat.

## 2. Results

### 2.1. Panobinostat Exhibits Dose-Dependent Cytotoxic Effects and Inhibits Cell Migration in GC Cell Lines

**Figure 1:**
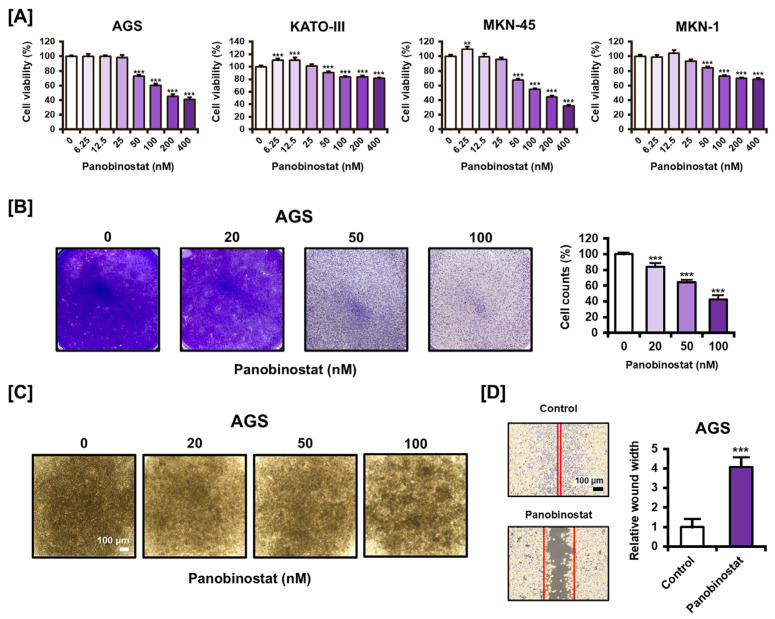
Effect of panobinostat on GC cell viability and proliferative capacity. (**A**) Cell viability of GC cell lines (AGS, KATO-III, MKN-45, and MKN-1) treated with increasing concentrations of panobinostat (0, 6.25, 12.5, 25, 50, 100, 200, and 400 nM) for 48 h, measured by CCK8 assay. (**B**) Crystal violet staining of AGS cells treated with panobinostat (0, 20, 50, and 100 nM) for 48 h. Stained cells were quantified using ImageJ software. (**C**) Spheroid formation of AGS cells treated with panobinostat (0, 20, 50, and 100 nM) for 72 h. Representative images are shown. (**D**) Wound-healing assay of AGS cells treated with panobinostat (100 nM) for 24 h. Relative wound width was quantified using ImageJ (version 1.53e). The red lines indicate the wound margins used for quantification of relative wound width. Data represent the mean ± standard deviation (SD) of three independent experiments. ** *p* < 0.01, *** *p* < 0.001 vs. control.

To investigate the therapeutic potential of panobinostat in GC, four human cell lines—AGS, KATO-III, MKN-45, and MKN-1—were treated with increasing concentrations of panobinostat ranging from 0 to 400 nM for 48 h. As shown in Figure 1A, panobinostat treatment resulted in a concentration-dependent reduction in cell viability across all tested cell lines, although the magnitude and pattern of response varied. AGS cells exhibited a consistent and gradual decrease in viability across the entire concentration range, displaying a well-defined dose–response relationship. In contrast, KATO-III and MKN-1 cells showed a more moderate reduction in viability, characterized by a relatively shallow dose–response curve at higher concentrations. Although MKN-45 cells were sensitive to panobinostat, their response at lower concentrations was less uniform compared with AGS cells.

Based on these observations, AGS cells were selected as the primary model for subsequent experiments. This selection was guided by the predictable and stable response profile of AGS cells, which provided an appropriate dynamic range for evaluating downstream molecular effects. The use of a model with a clear and reproducible dose–response relationship facilitated the assessment of combination treatment effects and supported the robustness of the mechanistic analyses presented in this study. Informed by the concentration-dependent effects observed in the cell viability assay, panobinostat concentrations for subsequent experiments were selected in AGS cells. For subsequent experiments, panobinostat was applied at concentrations of 0, 20, 50, and 100 nM. The 20 nM concentration was included to examine early molecular changes occurring prior to overt cytotoxicity, as cell viability was largely preserved at this dose. In contrast, 50 and 100 nM were chosen to capture dose-dependent biological effects associated with measurable reductions in cell viability.

Cell viability was also assessed using crystal violet staining (Figure 1B). A concentration-dependent decrease in viability was observed: at 100 nM, cell survival decreased to 42.47% relative to untreated controls. To examine the effects of panobinostat on three-dimensional cell growth, spheroid formation assays were performed using AGS cells (Figure 1C). Control spheroids (0 nM) maintained compact, spherical structures with defined borders. As panobinostat concentrations increased, spheroid morphology showed signs of progressive disruption. Observed changes included irregular edges, reduced cell cohesion, and looser cellular organization. At 100 nM, spheroids exhibited structural collapse and visible cell dispersion into the surrounding medium. Wound-healing assays were conducted using AGS cells to investigate panobinostat’s influence on cellular motility (Figure 1D). Following scratch formation, cells received panobinostat treatment or untreated control and were monitored over a 24 h period. Panobinostat-treated cells exhibited significantly impaired wound closure compared to control conditions, with gap widths approximately four-fold greater than those observed in untreated cells. These results indicate that panobinostat reduces the migratory capacity of AGS cells, in addition to its effects on cell viability and three-dimensional organization.

### 2.2. Panobinostat Induces G1 Cell Cycle Arrest and Apoptosis in AGS Cells

**Figure 2:**
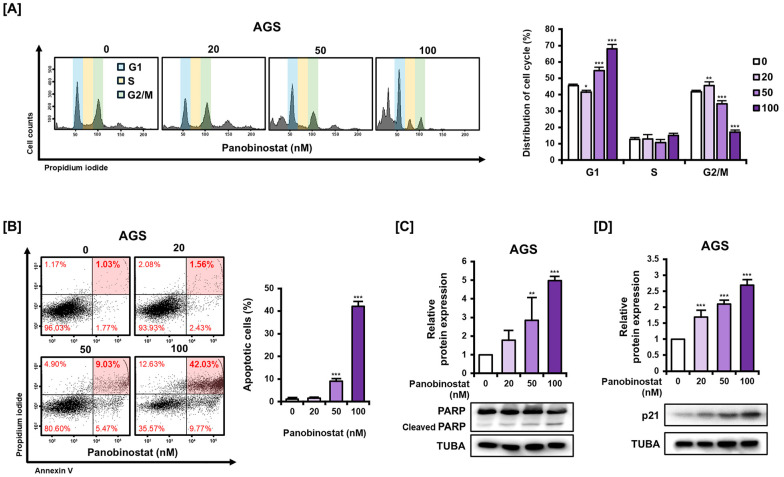
Effect of panobinostat on cell cycle progression and apoptosis in AGS cells. (**A**) Cell cycle distribution analyzed by flow cytometry after PI staining. AGS cells were treated with panobinostat (0, 20, 50, and 100 nM) for 24 h. (**B**) Apoptosis analysis by Annexin V/PI double staining and flow cytometry. AGS cells were treated with panobinostat (0, 20, 50, and 100 nM) for 48 h. The red shaded areas indicate late apoptotic cells (double-positive for Annexin V and PI), which were quantified. (**C**) Western blot analysis of PARP and cleaved PARP expression in AGS cells treated with panobinostat (0, 20, 50, and 100 nM) for 12 h. (**D**) Western blot analysis of p21 expression in AGS cells treated with panobinostat (0, 20, 50, and 100 nM) for 12 h. TUBA served as the loading control. Data represent the mean ± SD of three independent experiments. * *p* < 0.05, ** *p* < 0.01, *** *p* < 0.001 vs. control.

To determine whether panobinostat affects cell cycle progression in AGS cells, cells were treated with increasing concentrations of panobinostat and analyzed by flow cytometry following propidium iodide (PI) staining (Figure 2A). Treatment resulted in a dose-dependent accumulation of cells in the G1 phase, accompanied by a corresponding decrease in the G2/M population. An increase in the sub-G1 population was also observed, suggesting potential apoptotic induction following G1 arrest. To investigate panobinostat-induced apoptosis in greater depth, AGS cells were subjected to annexin V/PI double staining and analyzed by flow cytometry after treatment with increasing concentrations of the compound. A dose-dependent increase in apoptotic cells was detected, with apoptosis rates rising from 1.03% in the untreated control to 1.56%, 9.03%, and 42.03% with escalating concentrations of panobinostat (Figure 2B). As additional evidence of apoptosis, Western blot analysis revealed a gradual increase in cleaved PARP levels in a dose-dependent manner (Figure 2C), consistent with activation of apoptotic pathways. To link cell cycle arrest with the observed apoptotic response, we examined the expression of p21, a key regulator of G1 arrest and a well-established downstream effector of HDAC inhibition. Panobinostat treatment induced a robust and dose-dependent upregulation of p21 protein expression (Figure 2D). These results indicate that panobinostat-triggered p21 upregulation contributes to cell cycle blockade and subsequent apoptotic cell death in AGS cells.

### 2.3. Panobinostat Disrupts Mitochondrial Membrane Potential (MMP) in AGS Cells

**Figure 3:**
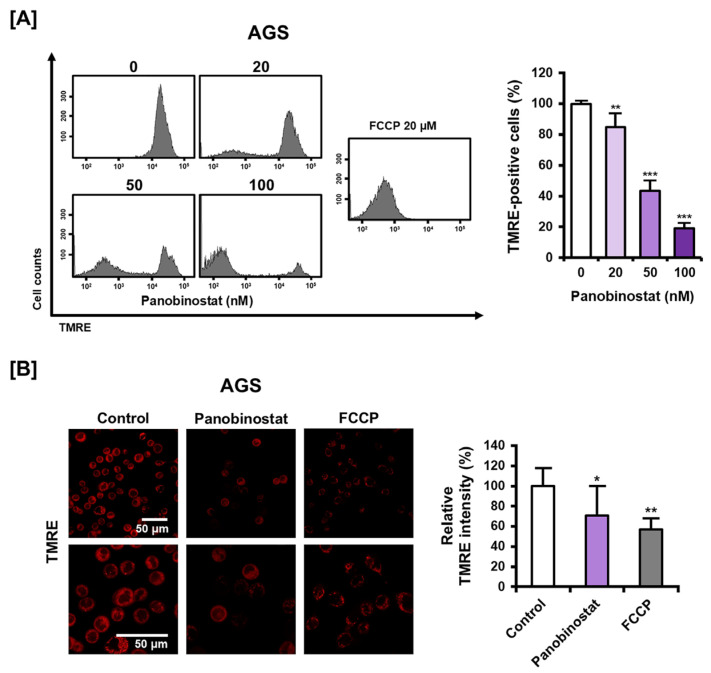
Effect of panobinostat on MMP in AGS cells. (**A**) Flow cytometric analysis of TMRE fluorescence in AGS cells treated with panobinostat (0, 20, 50, and 100 nM) or FCCP (20 μM, positive control) for 24 h. Cells were stained with TMRE (200 nM), and TMRE-positive cells were quantified. (**B**) Confocal microscopy images of TMRE-stained AGS cells under the same treatment conditions as in (**A**). Quantitative analysis of relative TMRE fluorescence intensity is shown. Data represent the mean ± SD of three independent experiments. * *p* < 0.05, ** *p* < 0.01, *** *p* < 0.001 vs. control.

The effect of panobinostat on MMP was assessed in AGS cells using tetramethylrhodamine ethyl ester (TMRE) staining followed by flow cytometry and confocal microscopy. Flow cytometric analysis demonstrated a dose-dependent reduction in TMRE fluorescence intensity, indicating a loss of MMP (Figure 3A). The mean fluorescence intensity decreased to 84.88%, 43.49%, and 18.89% at 20, 50, and 100 nM, respectively, relative to untreated control. This reduction was comparable to that induced by carbonyl cyanide 4-(trifluoromethoxy)phenylhydrazone (FCCP), a known mitochondrial uncoupler used as a positive control. Confocal microscopy further corroborated these findings, showing visibly reduced TMRE fluorescence in panobinostat-treated cells (Figure 3B). Quantitative analysis revealed that fluorescence intensity decreased to 70.93% of control levels following panobinostat treatment, similar to the 56.88% observed with FCCP. Collectively, these results indicate that panobinostat treatment leads to substantial loss of MMP in AGS cells.

### 2.4. Panobinostat Enhances 5-FU-Induced Cytotoxicity in AGS Cells

**Figure 4:**
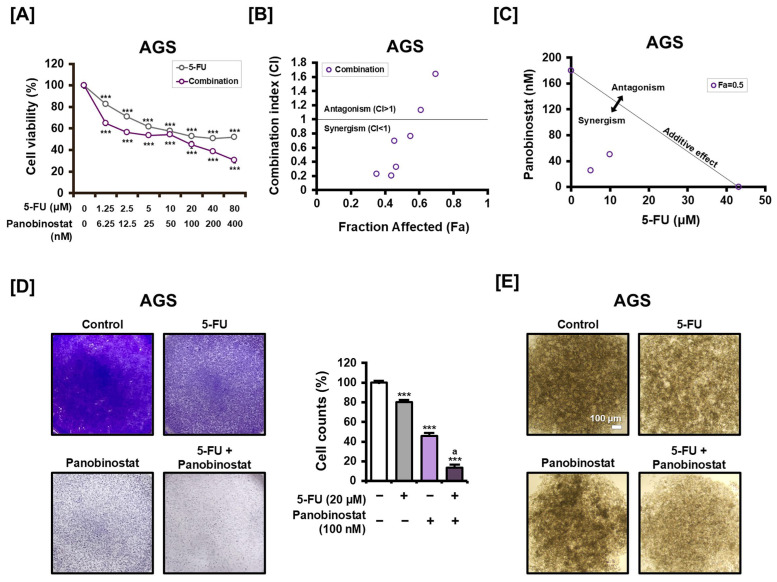
Effect of combination treatment with panobinostat and 5-FU on cell viability and spheroid growth in AGS cells. (**A**) Cell viability of AGS cells following treatment with 5-FU alone or in combination with panobinostat at a fixed 200:1 ratio for 48 h, determined by CCK8 assay. (**B**) Fa-CI plot generated using the Chou–Talalay method. CI values represent the degree of drug interaction, where CI < 1 indicates synergism, CI = 1 indicates an additive effect, and CI > 1 indicates antagonism. (**C**) Isobologram analysis for the combination of panobinostat and 5-FU at Fa = 0.5. The data points located below the line of additivity indicate a synergistic interaction between the two agents. (**D**) Crystal violet staining of AGS cells treated with 5-FU (20 μM), panobinostat (100 nM), or a combination of the two for 48 h. Stained cells were quantified using ImageJ software. (**E**) Spheroid formation of AGS cells treated as in (**D**) for 72 h. Representative images are shown. Data represent the mean ± SD of three independent experiments. *** *p* < 0.001 vs. control; a *p* < 0.05 vs. 5-FU alone.

Having demonstrated the monotherapeutic efficacy of panobinostat against GC cells, we then evaluated whether combination therapy with 5-FU could yield synergistic anticancer effects. AGS cells were treated with various concentrations of 5-FU, either alone or in combination with panobinostat. While 5-FU monotherapy induced a dose-dependent decrease in cell viability, co-treatment with panobinostat significantly further reduced viability across all tested concentrations (Figure 4A).

To quantitatively characterize this interaction, we performed a synergy analysis using the Chou–Talalay method. The fraction affected-combination index (Fa-CI) plot revealed a dose-dependent synergistic interaction, with CI values remaining consistently below 1.0 at low-to-moderate effect levels (Figure 4B). Potent synergism was observed at low-to-moderate effect levels (Fa < 0.5), where CI values consistently remained below 1.0. Notably, 100 nM panobinostat represents the threshold concentration at which synergistic interaction is maintained before transitioning toward antagonism at higher effect levels. The synergistic nature of this combination was further confirmed by the isobologram analysis at the 50% effect level (Fa = 0.5), where the combination data points were located well below the line of additivity (Figure 4C). To assess whether this chemosensitizing effect was restricted to AGS cells, additional combination experiments were performed in multiple GC cell lines, including KATO-III, MKN-45, and MKN-1. As shown in Figure S1, co-treatment with panobinostat consistently enhanced the cytotoxic effect of 5-FU across these models, despite differences in baseline drug sensitivity.

Consistent with these findings, crystal violet staining was performed. Treatment with 20 μM 5-FU alone reduced cell viability to 80.01% of the control levels, whereas the combination with 100 nM panobinostat decreased viability to 13.78% (Figure 4D). The spheroid formation assay also revealed that panobinostat enhanced 5-FU’s impact on three-dimensional cell architecture. Combination treatment disrupted spheroid integrity and cellular aggregation more effectively than 5-FU alone (Figure 4E). Collectively, these results show that panobinostat enhances the cytotoxic and anti-structural effects of 5-FU in AGS cells.

### 2.5. Panobinostat Enhances 5-FU-Induced Apoptosis in AGS Cells

**Figure 5:**
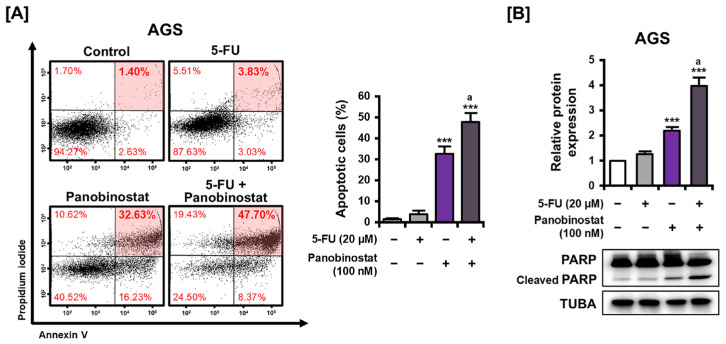
The effect of combined panobinostat and 5-FU treatment on apoptosis in AGS cells. (**A**) Apoptosis analysis involving Annexin V/PI double staining and flow cytometry. AGS cells were treated with 5-FU (20 μM), panobinostat (100 nM), or a combination of the two for 48 h. The red shaded areas indicate late apoptotic cells (double-positive for Annexin V and PI), which were quantified. (**B**) Western blot analysis of PARP and cleaved PARP expression in AGS cells was performed following treatment as shown in (**A**) for 12 h. TUBA served as the loading control. Data represent the mean ± SD of three independent experiments. *** *p* < 0.001 vs. control; a *p* < 0.05 vs. 5-FU alone.

Following on from the discovery that panobinostat enhanced 5-FU-induced cytotoxicity, we assessed whether the combination treatment also promoted apoptotic cell death. AGS cells were treated with 20 μM 5-FU alone or in combination with 100 nM panobinostat for 48 h, followed by Annexin V/PI double staining and flow cytometric analysis. As shown in Figure 5A, 5-FU monotherapy induced a slight increase in the proportion of late apoptotic cells (3.83%) compared to untreated controls (1.40%). In contrast, co-treatment with panobinostat markedly increased the proportion of late apoptotic cells to 47.70%, representing a statistically significant elevation compared to both the control and 5-FU alone. To further assess apoptotic signaling, cleaved PARP expression was assessed by Western blot analysis (Figure 5B). Minimal PARP cleavage was detected following 5-FU monotherapy, whereas the combination treatment led to a pronounced increase in cleaved PARP levels, consistent with enhanced apoptotic signaling. These results indicate that panobinostat enhances 5-FU-induced apoptotic responses in AGS cells.

### 2.6. Panobinostat Suppresses TS and Modulates Cell Cycle Regulators to Potentiate 5-FU Activity

**Figure 6:**
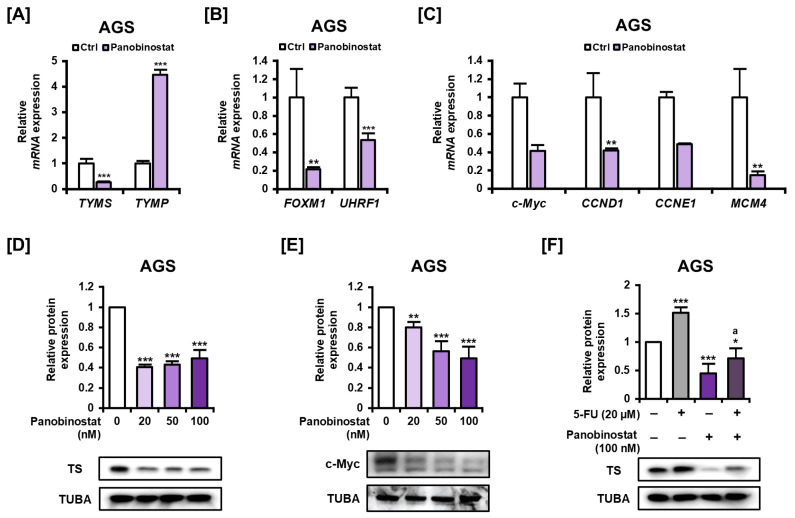
Panobinostat’s effect on the expression of genes related to 5-FU metabolism and cell proliferation in AGS cells. (**A**–**C**) qPCR analysis of mRNA expression levels of *TYMS* and *TYMP* (**A**), *FOXM1* and *UHRF1* (**B**), and *c-Myc*, *CCND1*, *CCNE1*, and *MCM4* (**C**) after treatment with panobinostat (20 nM) for 24 h. (**D**) Western blot analysis of TS protein levels after 12 h of treatment with panobinostat (0, 20, 50, and 100 nM). (**E**) Western blot analysis of c-Myc protein levels after 12 h of treatment with panobinostat (0, 20, 50, and 100 nM). (**F**) Western blot analysis of TS protein levels in AGS cells treated with 5-FU (20 μM), panobinostat (100 nM), or their combination for 12 h. TUBA served as the loading control. Data represent the mean ± SD of three independent experiments. * *p* < 0.05, ** *p* < 0.01, *** *p* < 0.001 vs. control; a *p* < 0.05 vs. 5-FU alone.

Having established that panobinostat in combination with 5-FU markedly reduces cell viability and enhances apoptotic cell death in AGS cells, we then investigated the underlying molecular basis for this synergistic effect. Given that TS is a critical determinant of 5-FU resistance, we focused on assessing changes in the expression of *TYMS* and other genes involved in 5-FU metabolism and cell proliferation. Quantitative PCR analysis revealed that panobinostat significantly downregulated *TYMS* mRNA expression while markedly upregulating *TYMP*, an enzyme involved in 5-FU activation and pyrimidine salvage pathways (Figure 6A). In addition, the expression of proliferation- and cell cycle-associated regulators, including *FOXM1* and *UHRF1* (Figure 6B), as well as *c-Myc*, *CCND1*, *CCNE1*, and *MCM4* (Figure 6C), was consistently suppressed, indicating broad inhibition of oncogenic transcriptional programs and cell cycle progression. At the protein level, Western blot analysis showed that panobinostat treatment reduced TS expression across all tested concentrations (Figure 6D), without a strict dose-dependent trend. In addition to assessing panobinostat’s impact on TS protein levels, we sought to determine its effect on other essential molecular targets. Consistent with the observed mRNA downregulation, panobinostat treatment led to a significant dose-dependent reduction in c-Myc protein expression (Figure 6E). Notably, while 5-FU monotherapy led to an increase in TS protein levels, this induction was effectively reversed by co-treatment with panobinostat (Figure 6F). Collectively, these findings indicate that panobinostat modulates the transcription of key genes regulating 5-FU metabolism and cell proliferation and reinforces TS suppression at the protein level, providing a mechanistic basis for its ability to potentiate the anticancer efficacy of 5-FU in GC cells.

## 3. Discussion

In this study, we demonstrate that panobinostat exerts potent monotherapeutic effects in GC cells and significantly enhances the anticancer efficacy of 5-FU when used in combination. HDACis, including panobinostat, are known for their pleiotropic antitumor activities, such as induction of apoptosis, inhibition of proliferation, and epigenetic remodeling in various solid tumors [22,23]. Our findings not only highlight the therapeutic potential of panobinostat as a chemosensitizer but also establish a mechanistic connection between HDAC inhibition and TS regulation.

Panobinostat monotherapy markedly reduced cell viability and spheroid formation while impairing cell migration and suppressing phenotypic traits associated with aggressive tumor behavior. These phenotypic changes were accompanied by G1 cell cycle arrest and a dose-dependent increase in the sub-G1 population, consistent with previous reports in other cancer models [24,25]. TMRE analysis confirmed the occurrence of mitochondrial membrane depolarization, implicating activation of the intrinsic apoptotic pathway. Furthermore, the expression of key transcriptional and epigenetic regulators (*FOXM1*, *UHRF1*, and *c-Myc*) was suppressed, aligning with HDACi-mediated transcriptional reprogramming. Specifically, c-Myc upregulates TS expression, thereby increasing the dNTP pool and significantly impacting pyrimidine synthesis [26]. Thus, panobinostat’s suppression of c-Myc directly disrupts the metabolic stability of GC cells.

Building on the observed monotherapeutic effects, we then explored the interaction between panobinostat and 5-FU. CI analysis indicated enhanced interaction between panobinostat and 5-FU within the evaluated range, suggesting a synergistic tendency rather than a purely additive effect. When combined with 5-FU, panobinostat induced a more pronounced reduction in cell viability and a stronger apoptotic response than either agent alone, supporting previous evidence that HDAC inhibition can potentiate DNA-damaging agents by disrupting chromatin structure, promoting DNA damage accumulation, and downregulating DNA repair genes [27,28]. Considering that resistance to 5-FU remains a major limitation in GC therapy, these results point to panobinostat as a promising adjuvant capable of overcoming chemoresistance through epigenetic reprogramming.

Interestingly, in the present study, treatment with 5-FU alone resulted in an increase in TS protein expression, even in non-resistant GC cells. This observation is consistent with previous reports describing compensatory upregulation of TS following 5-FU exposure [29]. These findings suggest that TS induction represents an early adaptive response to 5-FU treatment rather than a feature restricted to established resistant cell lines, highlighting the importance of TS regulation in determining fluoropyrimidine responsiveness. Consistent with this notion, previous studies have demonstrated that not only pharmacological inhibitors targeting TS, but also genetic suppression using siRNA or shRNA can significantly enhance tumor cell sensitivity to 5-FU, supporting the concept that reducing TS expression improves fluoropyrimidine efficacy [30,31]. In this context, panobinostat is of particular interest because it suppresses *TYMS* expression at the transcriptional level, potentially limiting the compensatory induction of 5-FU-triggered TS. This mode of regulation distinguishes panobinostat from conventional TS inhibitors that primarily act at the enzymatic level. At the same time, the enhanced efficacy observed with panobinostat and 5-FU is unlikely to be explained solely by TS suppression, reflecting widespread transcriptional reprogramming. These coordinated effects may collectively contribute to the pronounced sensitization to 5-FU and suggest that panobinostat-mediated chemosensitization extends beyond a single target pathway.

A notable aspect of our study is the integration of TS-axis gene regulation into the mechanistic framework of panobinostat’s activity in GC. While HDACis are broadly known to influence oncogenic signaling and transcriptional programs, their specific effects on the fluoropyrimidine metabolic pathway have been largely overlooked in GC. Our comprehensive gene expression analysis revealed functionally relevant changes in several key genes. *TYMS* was downregulated by panobinostat, whereas *TYMP*, which participate in 5-FU activation and pyrimidine salvage, was upregulated. Reduced *TYMS* expression limits dTMP availability, thereby enhancing 5-FU toxicity, a finding that is consistent with previous research [32]. Conversely, an increase in *TYMP* can facilitate the activation of 5-FU, potentially contributing to the observed synergy. However, compensatory or context-dependent effects cannot be excluded. Additionally, the quantities of *FOXM1* and *UHRF1*—which are critical regulators of transcription and epigenetic silencing—decreased, showcasing panobinostat’s capacity to reprogram epigenetic landscapes and potentially reverse drug resistance mediated by aberrant transcriptional control [33,34]. Furthermore, *c-Myc*, *CCND1*, *CCNE1*, and *MCM4*, which are involved in DNA replication and cell cycle progression, were also suppressed. This coordinated downregulation may lower the threshold for replication stress, sensitizing cells to S-phase-specific agents such as 5-FU [29]. While protein-level validation was performed for key regulatory nodes directly linked to the observed phenotypes, additional protein-level analyses of other transcriptionally altered targets would further refine the mechanistic framework.

To our knowledge, this is the first study to explore the intersection of HDAC inhibition and TS-pathway regulation in the context of GC. Our approach provides a mechanistic explanation for the observed synergistic effects and opens up new possibilities for biomarker-driven combination strategies. While previous studies have primarily focused on cell cycle arrest or histone acetylation levels [35], our inclusion of TS and related genes introduces an additional layer of translational relevance, especially considering the clinical use of TS expression as a predictor of fluoropyrimidine response [36,37].

Interestingly, both *DPYD*—a rate-limiting enzyme in 5-FU catabolism—and *MTHFR*, a modulator of folate metabolism influencing TS inhibition, were upregulated. Since both genes are typically associated with reduced fluoropyrimidine efficacy, this may appear paradoxical [38,39]. However, HDACi-induced transcriptional reprogramming often yields complex expression profiles that cannot be interpreted in isolation. The upregulation of *DPYD* and *MTHFR* might represent a compensatory cellular response to the extensive metabolic perturbations induced by panobinostat. Given the concurrent downregulation of *TYMS*, *TYMP*, and replication-related oncogenes, the net effect may still favor increased 5-FU sensitivity, suggesting that the overall metabolic context, rather than individual gene changes, determines therapeutic outcome.

## 4. Materials and Methods

### 4.1. Cell Culture and Treatment

The human GC cell lines AGS, KATO-III, MKN-45, and MKN-1 were purchased from the Korean Cell Line Bank (Seoul, Republic of Korea). The cells were maintained at 37 °C in a humidified atmosphere containing 5% CO_2_ in RPMI-1640 medium supplemented with 10% fetal bovine serum and 1% penicillin–streptomycin. During subculture, non-adherent dead cells and debris were removed from the supernatant. For KATO-III cells, which display both adherent and suspension growth characteristics, both fractions were collected, combined, and centrifuged. The resulting cell pellet was resuspended and used for further culture. Cells were detached using 0.25% trypsin.

Panobinostat (SML3060) and 5-FU (F6627) were purchased from Sigma-Aldrich (St. Louis, MO, USA). Panobinostat and 5-FU were dissolved in DMSO and added directly to the culture medium. To determine the individual effects of panobinostat, cells were treated with concentrations ranging from 0 to 500 nM. For combination studies, Panobinostat (100 nM) and 5-FU (20 μM) were added simultaneously.

### 4.2. CCK8 Assay

Cell viability was assessed using the Cell Counting Kit8 (CCK8) (ab228554, Abcam, Cambridge, UK) according to the manufacturer’s instructions. Cells were seeded into 96-well plates at a density of 5 × 10^4^ cells/mL in 100 μL of growth medium. After overnight incubation, cells were treated with specified concentrations of panobinostat and 5-FU and cultured for 48 h. Then, CCK8 solution was added to each well and incubated for an additional 2 h at 37 °C. Absorbance was measured at 450 nm using a Multiskan SkyHigh Microplate Spectrophotometer (Thermo Fisher Scientific, Waltham, MA, USA) to determine cell viability. Absorbance values were corrected by subtracting background absorbance from blank wells.

### 4.3. Crystal Violet Staining

Cells were seeded in 24-well plates at a density of 1 × 10^5^ cells/mL and allowed to adhere overnight. After attachment, cells were treated with Panobinostat in a dose-dependent manner (0, 20, 50, and 100 μM) or co-treated with 20 μM 5-FU and 100 nM panobinostat for 48 h. Following the treatment period, the culture medium was aspirated and cells were fixed with ice-cold methanol (−20 °C) for 10 min. The methanol was then removed, and the cells were stained with 0.5% crystal violet solution, which was prepared by diluting 1% crystal violet stock solution (V5265, Sigma-Aldrich) in distilled water. After 10 min of staining at room temperature, the staining solution was removed and the cells were washed twice with phosphate-buffered saline (PBS) and imaged. The proportion of viable cells was quantified using ImageJ software.

### 4.4. Spheroid Culture

Three-dimensional spheroid cultures were generated using the hanging drop method. Cells were suspended at a density of 1.6 × 10^6^ cells/mL in growth medium supplemented with the indicated concentrations of Panobinostat and 5-FU. Next, 25 μL of the cell suspension was dispensed in the form of individual droplets onto the inner surface of a 90 mm cell culture dish lid. To provide a humidification chamber, the bottom of the dish was filled with PBS. The lid was then inverted and placed over the dish, allowing the droplets to hang and promote spheroid formation via gravitational aggregation. Cultures were incubated at 37 °C in a humidified atmosphere with 5% CO_2_ for 72 h. After incubation, spheroids were imaged using an inverted phase-contrast microscope (CKX53, Olympus, Tokyo, Japan) equipped with a 4× objective lens. Images were processed using ToupView software (version 4.1; ToupTek, Hangzhou, China).

### 4.5. Wound-Healing Assay

Cell migration was evaluated using a wound-healing assay with culture inserts. Cells were seeded into μ-Dish 35 mm dishes containing 2-well culture inserts (80206, ibidi GmbH, Gräfelfing, Germany) and cultured until they reached over 90% confluence. After confluence, cells were treated with 100 nM panobinostat. The silicone inserts were then carefully removed using sterile tweezers to create a defined cell-free gap of approximately 500 μm. Following insert removal, cells were incubated for 24 h to allow migration. Images were captured using ToupView software. Cell migration was assessed by measuring the cell-free gap between wound edges at 24 h post-treatment. The mean wound width of the panobinostat-treated group was normalized to that of the control group, which was set to 1.0.

### 4.6. Cell Cycle Analysis

Cells were seeded into 60 mm culture dishes at a density of 1 × 10^5^ cells/mL and treated with 0, 20, 50, and 100 nM panobinostat for 24 h. Both adherent and floating cells were collected, washed with PBS, and fixed in 70% ethanol at 4 °C for at least 16 h. Fixed cells were resuspended in Annexin V binding buffer and stained with propidium iodide (PI) from the Annexin V FITC Apoptosis Detection Kit I (556547, BD Biosciences, Franklin Lakes, NJ, USA) and RNase A (R6148, Merck, Darmstadt, Germany) for 30 min in the dark. Flow cytometric analysis was performed using a BD FACS LSRFortessa flow cytometer (BD Biosciences) at the Ewha-Fluorescence Core Imaging Center, and data was acquired using BD FACSDiva software (version 8.0). A minimum of 10,000 events per sample were collected and cell cycle distribution was determined based on DNA content, as assessed by PI fluorescence intensity.

### 4.7. Annexin V and PI Staining

Apoptosis was assessed using the Annexin V FITC Apoptosis Detection Kit I according to the manufacturer’s instructions. Cells were cultured in 6-well plates at a density of 1 × 10^5^ cells/mL and treated with 0, 20, 50, or 100 nM Panobinostat, or a combination of 20 μM 5-FU and 100 nM Panobinostat for 48 h. Both floating and adherent cells were collected, washed once with PBS, and resuspended in Annexin V binding buffer. Then, cells were stained with Annexin V-FITC and PI in the dark for 15 min at room temperature. After staining, additional Annexin V binding buffer was added to each sample and the samples were transferred to FACS tubes for analysis. Flow cytometric analysis was performed using a BD FACS LSRFortessa flow cytometer. A minimum of 10,000 events per sample were collected, and the percentage of cells in late apoptosis (double-positive for both Annexin V and PI) was quantified using BD FACSDiva software.

### 4.8. MMP Analysis and Visualization

MMP was evaluated using TMRE staining followed by fluorescence-based quantification. FCCP served as a positive control to induce mitochondrial depolarization. For flow cytometric analysis, cells were seeded in 6-well plates at a density of 1 × 10^5^ cells/mL and treated with 0, 20, 50, or 100 nM panobinostat for 24 h. In the depolarization control group, 20 μM FCCP was added 10 min prior to TMRE staining. All samples were incubated with 200 nM TMRE in culture medium at 37 °C for 30 min in the dark, followed by washing with PBS. Fluorescence was measured using a flow cytometer (LSRFortessa, BD Biosciences, Franklin Lakes, NJ, USA), and MMP levels are expressed as percentages relative to untreated control cells. For confocal imaging, cells were seeded in confocal dishes (210350, SPL Life Sciences, Pocheon, Republic of Korea) and treated with 100 nM panobinostat for 24 h. TMRE staining was performed by incubating cells with 200 nM TMRE for 30 min at 37 °C in the dark under a humidified atmosphere. Then, 20 μM FCCP was added to the positive control group 10 min prior to TMRE staining. After the staining was completed, cells were gently washed with pre-warmed PBS and immediately imaged using a high-resolution laser scanning confocal microscope (LSM880 Airyscan, Carl Zeiss, Oberkochen, Germany). Fluorescence intensity was quantified using ImageJ software and normalized to the values of untreated control cells.

### 4.9. Quantitative Real-Time PCR

AGS cells were treated with 20 nM Panobinostat for 24 h. Total RNA was extracted using the AccuPrep Universal RNA Extraction Kit (K-3140, Bioneer, Daejeon, Republic of Korea), and RNA concentration and purity were measured using a Multiskan SkyHigh Microplate Spectrophotometer. Complementary DNA was synthesized from 1 μg of total RNA using AccuPower RT PreMix (K-2261, Bioneer). Quantitative real-time PCR was performed on a QuantStudio 3 Real-Time PCR System (A28566, Thermo Fisher Scientific) using AccuPower^®^ 2X GreenStar™ qPCR Master Mix (K-6252, Bioneer). The mRNA expression levels of *TYMS*, *TYMP*, *FOXM1*, *UHRF1*, *c-Myc*, *CCND1*, *CCNE1* and *MCM4* were analyzed using the gene-specific primers listed in Table 1. Relative mRNA expression levels were normalized to *GAPDH* and calculated using the 2^−ΔΔCT^ method.

### 4.10. Western Blot Analysis

Protein expression was analyzed by Western blotting. Total proteins were extracted using RIPA buffer (PB.RB500, Allforlab, Seoul, Republic of Korea) supplemented with protease inhibitor cocktail (P8340, Merck), and protein concentrations were determined using the Bradford assay (J61522, Alfa Aesar, Haverhill, MA, USA). Equal amounts of protein were separated on 10% SDS–polyacrylamide gels and transferred to nitrocellulose membranes (0.45 μm; BR1620115, Bio-Rad, Hercules, CA, USA). Membranes were blocked with 2% BSA (BSAS-0.1, Bovogen, Keilor East, VIC, Australia) in TBS-T containing 0.1% Tween-20 (HC0777, Fourbio, Seoul, Republic of Korea) for 1 h at room temperature, followed by incubation with primary antibodies overnight at 4 °C. After washing, membranes were incubated with secondary antibodies diluted in 2% skim milk/TBS-T for 1 h at room temperature. Protein bands were visualized using a KwikQuant Pro Imager (D1010, Kindle Biosciences, Greenwich, CT, USA) and analyzed with KwikQuant Image Analyzer software (v5.9). Band intensities were normalized to α-tubulin. Primary antibodies were obtained from Cell Signaling Technology (Danvers, MA, USA) and ABclonal Korea (Seongnam, Republic of Korea).

### 4.11. Statistical Analysis

All quantitative data were obtained from at least three independent biological experiments, and results are expressed as the mean ± SD. Data distribution was assessed prior to statistical analysis and statistical comparisons were performed using parametric tests as appropriate. Statistical analyses were performed using SAS software (version 9.4; SAS Institute, Cary, NC, USA). Statistical significance was defined as * *p* < 0.05, ** *p* < 0.01, and *** *p* < 0.001. In co-treatment experiments, comparisons between 5-FU alone and co-treatment groups were marked with “a” (*p* < 0.05).

## 5. Conclusions

**Figure 7:**
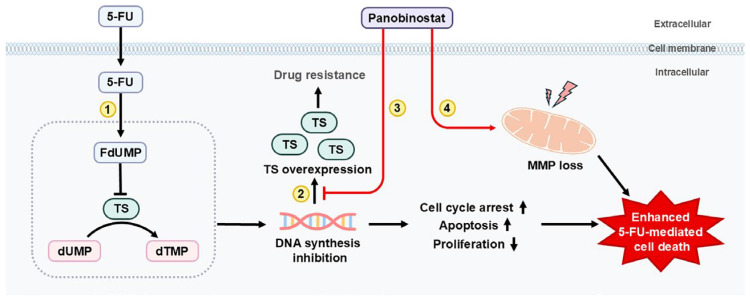
The proposed mechanism by which panobinostat enhances 5-FU-mediated cytotoxicity in GC cells. 5-FU is metabolized to FdUMP, which inhibits TS and disrupts dTMP synthesis; however, compensatory TS overexpression contributes to acquired drug resistance. Panobinostat suppresses basal and 5-FU-induced TS expression, leading to enhanced inhibition of DNA synthesis. In parallel, panobinostat induces MMP loss, promotes cell cycle arrest, and activates apoptosis, synergistically enhancing 5-FU-mediated cancer cell death.

In conclusion, our findings demonstrate that panobinostat not only exerts intrinsic anticancer effects in GC cells but also functions as a potent chemosensitizer that overcomes 5-FU resistance through a TS-associated mechanism. Panobinostat suppresses both basal and 5-FU-induced TS expression, disrupts MMP, and promotes cell cycle arrest and apoptosis, amplifying 5-FU-mediated cytotoxicity. Although TS regulation appears to be a key contributing mechanism, the effects of panobinostat are likely influenced by broader transcriptional and cell cycle regulatory pathways. These integrated molecular and cellular effects are summarized schematically in Figure 7. This mechanistic framework supports the rational combination of panobinostat and 5-FU as a promising strategy for overcoming chemoresistance in GC. An important limitation of the present study is that the combination strategy was evaluated primarily in treatment-naïve GC cell models. Since acquired resistance to 5-FU often involves compensatory upregulation of TS and broader metabolic reprogramming, future studies employing established 5-FU-resistant GC cell lines will be critical for determining whether panobinostat can overcome resistance-associated adaptations. Moreover, investigations incorporating broader validation of p53-dependent and mitochondrial apoptotic regulators will be important to further refine the mechanistic framework proposed here. Taken together, our findings suggest that panobinostat may have value as a potential chemosensitizing agent in fluoropyrimidine-based therapy for GC. Further studies in resistant models and in vivo systems will be required to establish its clinical relevance.

## Figures and Tables

**Table 1:** Primer sequences for RT-qPCR analysis.

Gene	Forward Primer	Reverse Primer
*GAPDH*	5′-TCGTGGAAGGACTCATGACC-3′	5′-ATGATGTTCTGGAGAGCCCC-3′
*TYMS*	5′-CCAAAGCTCAGGATTCTTCG-3′	5′-AGTTGGATGCGGATTGTACC-3′
*TYMP*	5′-CAATGATCAGCGGACGTGG-3′	5′-ACTCTGACCCACGATACAGC-3′
*FOXM1*	5′-GGTACCTATCCAGTTCCCGG-3′	5′-TCTGAGCTCATGAGGGAAGC-3′
*UHRF1*	5′-GGTTGTGAAATACTGGCCCG-3′	5′-TTCTTGATCCGGTCCTTCCC-3′
*c-Myc*	5′-CCCTCAACGTTAGCTTCACC-3′	5′-CAGCAGCTCGAATTTCTTCC-3′
*CCND1*	5′-TGCTGGTTTTCTACCCAACG-3′	5′-AGTGCTTGGAAATGGAATGG-3′
*CCNE1*	5′-GGGGAGCTCAAAACTGAAGC-3′	5′-ACATGGCTTTCTTTGCTCGG-3′
*MCM4*	5′-GTTCACCACTGACATACGGC-3′	5′-CAGACTGCAGATCCACTTGC-3′

## Data Availability

The original contributions presented in this study are included in the article/Supplementary Materials. Further inquiries can be directed to the corresponding author.

## Supplementary Materials

The following supporting information can be downloaded at https://www.mdpi.com/article/10.3390/ijms27031516/s1.

## Abbreviations

The following abbreviations are used in this manuscript:

5-FU	5-fluorouracil
GC	gastric cancer
TS	thymidylate synthase
HDACi	histone deacetylase inhibitor
HDAC	histone deacetylase
SAHA	suberoylanilide hydroxamic acid
PI	propidium iodide
MMP	mitochondrial membrane potential
TMRE	tetramethylrhodamine ethyl ester
FCCP	carbonyl cyanide 4-(trifluoromethoxy)phenylhydrazone
PBS	phosphate-buffered saline
