# Supplementary material for: Panobinostat Potentiates the Antitumor Efficacy of 5-Fluorouracil in Gastric Cancer by Suppressing Thymidylate Synthase Expression
Source: Int J Mol Sci. 2026 Feb 3;27(3):1516. doi: 10.3390/ijms27031516 (PMC12898101; doi:10.3390/ijms27031516)
Supplement: Supplementary file 1 [file ijms-27-01516-s001.zip › ijms-4078173-supplementary.pdf]

Panobinostat potentiates the antitumor efficacy of 5-fluorouracil in gastric cancer by suppressing thymidylate synthase expression

Sooyeon Park [1,†], Nayeon Kim [1,†] and Changwon Yang [1,*]

[1] Department of Science Education, Ewha Womans University, Seoul 03760, Republic of Korea
* Correspondence: cyang@ewha.ac.kr; Tel.: +82-2-3277-2688
† These authors contributed equally to this work.

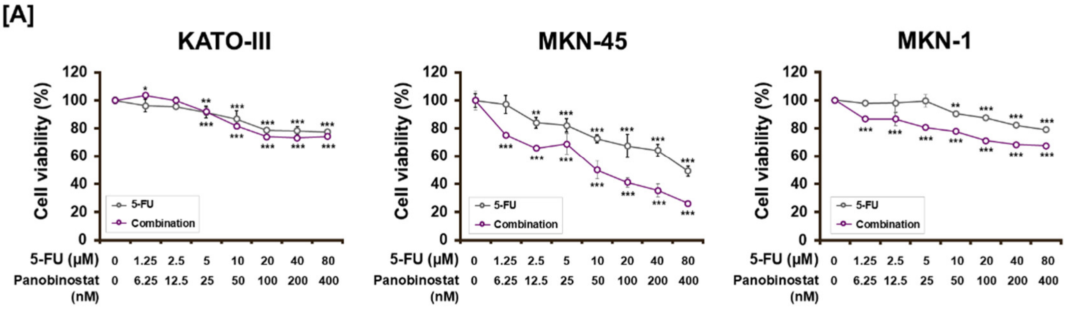

Figure S1. Effect of combination treatment with panobinostat and 5-FU on cell viability in gastric cancer cell lines. Cell viability of KATO-III, MKN-45, and MKN-1 cells treated with increasing concentrations of 5-FU (0, 1.25, 2.5, 5, 10, 20, 40, and 80 μM) either alone or in combination with panobinostat at a fixed 200:1 ratio (corresponding panobinostat concentrations: 0, 6.25, 12.5, 25, 50, 100, 200, and 400 nM) for 48 h, as measured by CCK8 assay. Data represent the mean ± SD of three independent experiments. *$p < 0.05$, **$p < 0.01$, ***$p < 0.001$ compared with 5-FU alone at the corresponding concentration.
